# Intravesical repair of vesicovaginal fistula guided by cystoscopy

**DOI:** 10.52054/FVVO.13.2.022

**Published:** 2021-06-28

**Authors:** M.A. Tavares, S Campagne Lpiseau, M Canis, R Botchorishvili

**Affiliations:** Department of Gynecology and Obstetrics, CHU de Clermont Ferrand, 63100 Clermont-Ferrand, France; Centre International de Chirurgie Endoscopique (CICE), 63000 Clermont-Ferrand, France; Serviço de Ginecologia e Obstetrícia, Hospital Vila Franca de Xira, 2600-009 Vila Franca de Xira, Portugal

**Keywords:** Vesicovaginal fistula, 3 mm laparoscopy, transvesical approach, cystoscopy, recurrent fistula

## Abstract

**Background:**

Vesicovaginal fistulas (VVF) are an unusual problem that may significantly affect a patient’s quality of life. The main causes for this condition are labour complications (mostly in developing countries) and pelvic surgeries (in industrialised countries). Treatment may be conservative or surgical. Regarding surgical treatment, there is still debate about the best approach and surgical technique.

**Objective:**

To demonstrate a correction of a VVF guided by cystoscopy using intravesical laparoscopic instruments.

**Methods:**

Case report and surgical video of a recurrent VVF treated with a hybrid technique involving direct transvesical insertion of 3 mm laparoscopic trocars and instruments guided by cystoscopy. As far as we know, although there are some reported techniques that use a combination of transvesical laparoscopic instruments and cystoscopy, this is the least invasive and most ergonomic technique described.

**Results:**

Two years after surgery, the patient remains asymptomatic and with no fistula recurrence.

**Conclusion:**

The transvesical approach guided by cystoscopy seems to be an effective, safe and ergonomic minimally invasive procedure for VVF repair.

## Introduction

A vesicovaginal fistula (VVF) is an abnormal tract that forms between the vagina and the bladder causing continuous urine leak through the vagina. It may have a significant impact on the quality of life of the patient affecting medical well-being, psychologic status as well as sexual and social life. Vesicovaginal fistulas are an uncommon problem that occur in 0.5-2.0% of all pelvic surgeries (Nerli et al., 2019). In developing countries 97% of all VVF are secondary to prolonged obstructed labour whereas in industrialised countries the majority of cases present after gynaecological or urological surgery. (Bodner-Adler et al., 2017; Nerli et al., 2019). Other risk factors include pelvic irradiation, endometriosis, malignancy, anatomical distortion by myomas or ovarian tumors, impaired healing status and infection (Nerli et al., 2019).

Both conservative and surgical treatments are available for the management of VVF. However, there is still no consensus regarding the best management of this complication. Conservative treatments may be appropriate as a primary attempt in small (<10mm), clean, non-malignant fistulas (Bodner-Adler et al., 2017) with a described success rate of 5-11% (Breen and Ingber, 2018).

Surgical treatment has a success rate of 75 to 95% (Bodner-Adler et al., 2017). Many surgical techniques have been described to treat VVF with various surgical routes (laparotomy, transvaginal, laparoscopy, robotic-assisted surgery and other less common minimal-invasive techniques). Regarding minimal-invasive techniques, some case series have reported the utilisation of a hybrid technique using cystoscopy and intravesical treatment with favorable outcomes (McKay, 2001; Nerli and Reddy, 2010). We present a case report with a surgical video and an overview of literature regarding this surgical approach.

## Case report

A 32-year-old-female, G1P1, presented to our department after the diagnosis of a VVF. Her past history revealed three previous surgeries for deep endometriosis, the last one performed three months before the appointment and involving resection of an endometriotic vesical nodule, partial cystectomy, reimplantation of the left ureter and a JJ stent placement. Although the patient described occasional urinary leakage, this symptom was assumed to be due to the JJ catheter. However, after JJ stent removal, a cystoscopy was performed and a 2mm fistula was diagnosed. The patient was submitted to a transvaginal repair of the VVF, but this procedure proved very difficult for the fistula was in a very high position (about 8cm from the hymenal plane) and, consequentially, problematic to see and approach vaginally. A laparoscopic approach was tried but, due to the extensive adhesions and anatomical distortion it was also impossible to correct the repair by this route. Thus, the surgeons decided to correct the small defect using a vaginal procedure. Although this improved the patient’s symptomatology, she still experienced some occasional urine leakage and thereby, three months after the first VVF repair, she was submitted to a second vaginal procedure. Seven months later, the patient symptoms persisted and her CT-Urogram revealed a persistent VVF located next to the ureterovesical implantation site. Being a recurrent VVF, a new approach was considered. The patient was informed and advised about this new technique and signed an informed consent form for the surgery and for the case publication.

## Surgical Procedure

The procedure was performed under general anesthesia and the patient was placed in the lithotomy position. A swab was introduced in the vaginal extremity of the fistula exiting into the bladder. Cystoscopy was performed with visualisation of the vaginal swab and confirmation of the fistula’s opening at the posterior vesical wall, outside and distant from the right ureteral ostium.

Two 3 mm suprapubic trocars (KARL STORZ SE & Co. KG, Tuttlingen, Germany) were introduced in each side of the midline just above the pubic symphysis and into the bladder under direct cystoscopic control. A 4 mm, 70-degree cystoscope charr 22 was used (KARL STORZ SE & Co. KG, Tuttlingen, Germany) and bladder distention was achieved with physiological serum.

Surgical instruments were introduced into the suprapubic trocars and manipulated by the surgeon while the cystoscopic camera was managed by the assistant.

Mucosal and muscular excision of the bladder tissues surrounding the fistula was performed using a dissector and scissor. Fistula closure was accomplished with a two-layer suture with extracorporeal knots using 3/0 Biosyn™ Monofilament Absorbable Sutures. As 3 mm trocars do not allow curved-needle introduction, it was necessary to bend the needle in order to introduce/ remove it from the bladder. Suprapubic trocars were removed without the need of sutures.

There were no intra or post-operative complications. The patient was catheterised for three weeks and maintained antibiotic therapy for seven days. Two years after surgery, the patient is asymptomatic and with no recurrence of the VVF.

## Discussion

VVF are still a challenge to treat and guidelines for their management are lacking. Recurrence rates (15%) are higher in large (>10mm), multiple or complex fistulas caused by obstetric complications and with reported urinary tract infections prior to treatment (Nerli et al.,2019). Factors that improve the repair rate of a VVF include adequate pre- operative evaluation, large exposure of the fistula and its surrounding tissues with excision of the fibrosed tissue, tension-free closure, avoiding excessive tension on the vaginal mucosa, maintenance of an uninfected and dry suture. Bladder closure appears to be more relevant than vaginal closure to accomplish a favourable outcome (Ayed et al., 2006).

Transvaginal treatment is the most commonly reported surgical approach for VVF repair. It is associated with shorter operative as well as hospitalisation time and reduced blood loss. However, it has an increased risk of vaginal shortening and it may be difficult to adequately expose some fistulas and suture them correctly (Nerli and Reddy, 2010). Endoscopic techniques are more favourable than laparotomy as they are associated with better visualisation, shorter hospital stays, faster recovery, less postoperative pain and better cosmetic results.

As for cystoscopic-guided procedures, literature review revealed some case series describing different techniques. Okamura et al. (1997) described a case of an endoscopic transvesico- transurethral approach for the repair of VVF. The author used a cystoscopy for trocar placement (two 5-mm suprapubic trocars) but then changed to a 0º optic in the suprapubic position. Fistula closure was achieved using a needle-holder in a suprapubic trocar and another needle-holder via urethral route.

McKay reported 6 cases of transurethral suture cystorrhaphy for repair of vesicovaginal fistulas. The author perfected his technique achieving a procedure that uses a single 5-mm suprapubic arthroscope and a self-righting laparoscopic needle driver inserted via urethral route. Akkoc et al. (2018) described 9 cases of transvesicoscopic bipolar sealing of VVF using cystoscopy as a camera and two suprapubic trocars of 5-mm for instruments. Guntaka et al. (2014) and Nerli et al. (2019) published a series of 7 cases of transvesicoscopic repair of VVF. Cystoscopy guidance was initially used for trocar introduction (three 5-mm ports) but then the cystoscope was withdrawn and an optic was introduced in one of the three suprapubic trocars. Grange et al. (2014) presented 9 cases of VVF repair by vesicoscopy (percutaneous cystoscopic surgery). The authors distended the bladder by insufﬂ ating CO 2 through a foley catheter and then introduced three 5 mm suprapubic ports under direct vision with a 0º optic. A 30º optic was used for the rest of the procedure. The bladder was suspended to the anterior abdominal wall with sutures and the VVF were excised using an ultrasonic device. Both the bladder and vaginal defects were closed with sutures. Using three 5 mm suprapubic ports limits the distance between instruments and therefore, surgical ergonomy. Transvesicoscopic VVF repair has also been performed using a laparoendoscopic single-site technique through the bladder (Roslan et al., 2012). In this case, a single 15 mm suprapubic incision was made to insert the single port device which would accommodate one 10 mm 30º optic and three 5 mm instruments. Bladder distention was achieved with CO2 insufflation. Excision of the fistula was made using monopolar energy and the fistula’s closure was performed using a barbed suture, therefore avoiding the need for tying knots.

Although the use of gas as a distention medium is more widespread for transvesicoscopic procedures (Grange et al., 2014) then water irrigation, they are equally safe and effective (Matthews et al., 1983). As the surgical team was more familiar with a liquid distension approach, this was the chosen medium to distend the bladder.

Energy devices such as monopolar, bipolar or ultrasonic (Roslan et al., 2012; Grange et al., 2014;, Akkoç et al., 2018) may be used to excise the fistulous tract. However, in this particular case, there was no need to use a energy device and a scissor and dissector were sufficient to excise the fibrotic and devitalised tissue without creating any energy effect on the surrounding tissue. In general, transvesicoscopic techniques own the advantages of minimal invasive surgery but with decreased risk of trauma to adjacent organs, less postoperative ileus and less bleeding (Nerli and Reddy, 2010).

We present a case that may be an evolution from the previous described techniques. Cystoscope guidance allows the introduction of only two suprapubic trocars maintaining the ergonomic and surgical movements of a normal laparoscopy instead of using a suprapubic and a transurethral instrument. In addition, 3 mm trocars and instruments cause less tissue trauma, less pain and better cosmetic results than traditional 5 mm trocars without increasing the complication rate. However, the need to bend the needle before suturing (to pass in the 3 mm trocars) can lead to some safety issues, such as needle breaking, that should be monitored during the procedure.

Although not performed in this case, bladder suspension to the anterior wall may be important to avoid bladder collapse in case of accidental trocar withdrawal (Grange et al., 2016)

This technique allowed, in addition to the previous described advantages, direct visualisation of the fistula ostium inside the bladder, which permitted a safe dissection and suture of the fistula, without compromising the ureter and with no need for ureteral stents introduction.

## Conclusion

Although further studies are needed, the transvesical approach guided by cystoscopy seems to be an effective, safe, minimal-invasive procedure for VVF repair. Adding to the already known advantages of minimal-invasive surgery, this technique with only two suprapubic 3-mm trocars causes less and smaller scars compared to the previous described techniques and allows maintenance of ergonomics as in laparoscopy. With the development and improvement of surgical instruments and laparoscopic techniques, it is possible for an experienced surgeon to explore new surgical approaches, especially in difficult cases such as recurrent VVF
